# ﻿Characterization of two new *Pylorgus* mitogenomes (Hemiptera, Lygaeidae, Ischnorhynchinae) and a mitochondrial phylogeny of Lygaeoidea

**DOI:** 10.3897/zookeys.1166.104103

**Published:** 2023-06-08

**Authors:** Cuiqing Gao, Wen Dong

**Affiliations:** 1 Co-Innovation Center for Sustainable Forestry in Southern China, College of Forestry, Nanjing Forestry University, Nanjing, Jiangsu 210037, China Nanjing Forestry University Nanjing China

**Keywords:** Heteroptera, mitochondrial DNA, next-generation sequencing, phylogenetic analysis, *Pylorgusporrectus*, *Pylorgussordidus*

## Abstract

Lygaeidae is a large family of Hemiptera (Heteroptera) currently separated into three subfamilies, Ischnorhynchinae, Lygaeinae, and Orsillinae. In this research, the complete mitogenomes of the iscnorhynchines *Pylorgusporrectus* Zheng, Zou & Hsiao, 1979 and *Pylorgussordidus* Zheng, Zou & Hsiao, 1979 were sequenced, and the phylogeny of *Pylorgus* and the Lygaeidae with known complete mitogenomes were examined. The mitogenomes are 15,174 bp and 15,399 bp in size, respectively, and comprised of 13 protein-coding genes (PCGs), 22 transfer RNA genes (tRNAs), two ribosomal RNA genes (rRNAs), and a control region (D-loop). Nucleotide composition is biased toward A and T, and the gene order is identical to that of the putative ancestral arrangement of insects. Eleven PCGs begin with a typical ATN, and the remaining two PCGs begin with TTG (*cox1* and *nad4l*). All tRNAs had a typical cloverleaf secondary structure, but some of them had individual base mismatches. The phylogenetic analyses based on the concatenated nucleotide sequences of the 13 PCGs, using Bayesian inference and maximum likelihood, support the monophyly of Lygaeidae. The results show that *P.porrectus* and *P.sordidus* clustered with nine other Lygaeidae. This study includes the first complete sequencing of the mitochondrial genomes of two *Pylorgus* species, which will provide important data for studying the phylogenetic position of Lygaeidae in Lygaeoidea and reconstructing the phylogenetic relationships within Pentatomomorpha.

## ﻿Introduction

The Lygaeoidea represents the second largest superfamily within the infraorder Pentatomomorpha and includes over 4660 described species in 16 families (Henry 2017; Dellapé and Henry 2020). Most Lygaeoidea feed mainly on mature seeds (Schuh and Slater 1995); although Blissidae, Colobathristidae, Malcidae, and Piesmatidae predominantly feed on plant sap (Sweet 2000; Henry et al. 2015), Berytidae are mostly phytophagous, with a few becoming pests, although some have been shown to be predatory (Henry 2000), and Geocoridae are primarily predators but sometimes also feed on seeds and leaves of plants (Sweet 2000).

Currently, three subfamilies of Lygaeidae (sensu stricto) are recognized: Ischnorhynchinae, Lygaeinae, and Orsillinae (Dellapé and Henry 2020). The main diagnostic characters of Lygaeidae are as follows: bucculae well developed, pronotal calli with an impressed transverse groove, scutellum usually with a raised cross-shaped carina, and hamus present on wings. Abdominal spiracles on segments II to VII dorsal (Malipatil et al. 2020).

To date, the phylogeny of Lygaeidae is unresolved (Yao et al. 2012; Zhang et al. 2019), and the status of Orsillinae and Ischnorhynchinae in relation to Lygaeidae (sensu stricto) continues to be discussed. Henry (1997) proposed that Orsillinae and Ischnorhynchinae be classified as subfamilies of Lygaeidae. However, Sweet (2000) recognized them as separate families from the Lygaeidae (Orsillidae and Ischnorhynchidae). A few workers have followed Sweet in adopting the family Orsillidae (Eyles and Malipatil 2010; Malipatil 2010; Ge and Li 2019), whereas Henry et al. (2015), supported by Schuh and Weirauch (2020), disagreed with Sweet, who provided no evidence to support his hypothesis.

The complete mitochondrial genome data of nine species in Lygaeidae are included on NCBI, and only two species of Ischnorhynchinae. However, for the largest genus in this subfamily, *Pylorgus*, the mitochondrial genome data is totally unknown. Therefore, in the present study, we obtained the complete mitochondrial genomes of two *Pylorgus* species, *Pylorgusporrectus* Zheng, Zou & Hsiao, 1979 and *Pylorgussordidus* Zheng, Zou & Hsiao, 1979, by using the next-generation sequencing technology. Furthermore, we constructed the phylogenetic trees based on the mitogenomes of 21 species of the superfamily Lygaeoidea and four outgroup species, which will provide important data for further studies on the phylogenetic position of Lygaeidae in Lygaeoidea and be also useful to reconstruct the phylogenetic relationships within Pentatomomorpha.

## ﻿Materials and methods

### ﻿Sample collection, DNA extraction, and mitogenome sequencing

**Figure 1.**
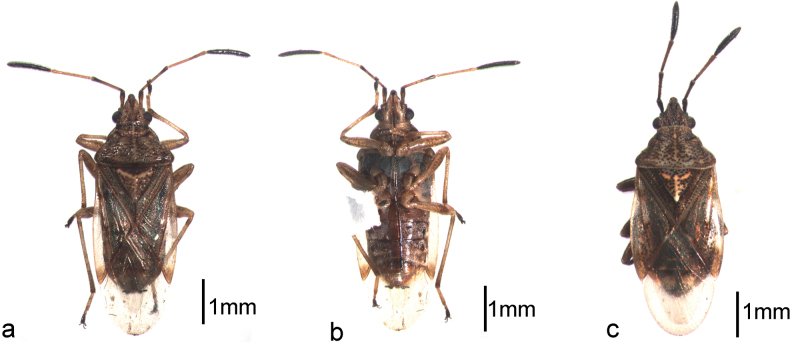
*Pylorgus* species sequenced **a, b***P.porrectus*, dorsal and ventral views **c***P.sordidus*, dorsal view.

Adults of *Pylorgusporrectus* (Fig. 1a, b) were collected from Zhongshan Botanical Garden (32°03.27'N, 118°49.85'E), Nanjing, Jiangsu Province, China, 20 April 2022, Cuiqing Gao leg. Adults of *P.sordidus* (Fig. 1c) were collected from Hongqi Management and Protection Station, Yintiaoling Nature Reserve (31°23.87'N, 109°41.32'E), Wuxi County, Chongqing, China, 1 July 2022, Suyan Cao leg. The specimens were identified based on the morphological characteristics seen under a Zeiss Stereo Discovery V8 Zoom Microscope and deposited in the Insect Collection, College of Forestry, Nanjing Forestry University.

**Table 1.** Species used in this study.

Family	Subfamily	Species	Length (bp)	GenBank No.
Lygaeidae	Ischnorhynchinae	*Kleidocerysresedae* (Panzer, 1797)	14,688	KJ584365.1
	Ischnorhynchinae	*Pylorgusporrectus* Zheng, Zou & Hsiao, 1979	15,174	OP793792
	Ischnorhynchinae	*Pylorgussordidus* Zheng, Zou & Hsiao,1979	15,399	OQ064783
	Ischnorhynchinae	*Crompusoculatus* Stål, 1874	15,332	MW619652.1
	Lygaeinae	*Arocatusmelanocephalus* (Fabricius, 1798)	15,409	NC_063142.1
	Lygaeinae	*Tropidothoraxcruciger* (Motschulsky, 1859)	15,781	NC_056293.1
	Lygaeinae	*Tropidothoraxsinensis* (Reuter, 1888)	15,422	MW547017.1
	Orsillinae	*Nysiusplebeius* Distant, 1883	17,637	MN599979.1
	Orsillinae	*Nysiuscymoides* (Spinola, 1837)	16,301	MW291653.1
	Orsillinae	*Nysiusfuscovittatus* Barber, 1958	14,575	NC_050167.1
	Orsillinae	*Nithecusjacobaeae* (Schilling, 1829)	14,206	MW619651.1
Berytidae	Metacanthinae	*Yemmalysusparallelus* Stusak, 1972	15,747	NC_012464.1
	Metacanthinae	*Metatropislongirostris* Hsiao, 1974	15,744	NC_037373.1
Blissidae		*Bochrusfoveatus* Distant, 1879	14,738	NC_065814.1
		*Capodemussinuatus* (Slater, Ashlock & Wilcox, 1969)	15,199	NC_065815.1
Geocoridae	Geocorinae	*Geocorispallidipennis* (Costa, 1843)	14,592	NC_012424.1
	Henestarinae	*Henestarishalophilus* (Burmeister, 1835)	14,868	MW619656.1
Malcidae	Chauliopinae	*Chauliopsfallax* Scott, 1874	15,739	NC_020772.1
	Malcinae	*Malcusinconspicuous* Štys, 1967	15,575	NC_012458.1
Rhyparochromidae	Rhyparochrominae	*Neolethaeusassamensis* (Distant, 1901)	15,067	NC_037375.1
	Rhyparochrominae	*Bryanellocorisorientalis* Hidaka, 1962	15,606	NC_063139.1
Pyrrhocoridae		*Dysdercusevanescens* Distant, 1902	15,635	MW619727.1
Coreidae	Hydarinae	*Hydaropsislongirostris* (Hsiao, 1963)	16,521	EU427337.1
Rhopalidae		*Aeschyntelusnotatus* Hsiao, 1963	14,532	EU427333.1
Alydidae		*Riptortuspedestris* (Fabricius, 1775)	17,191	EU427344.1

The complete genomic DNA was extracted from an adult sample using a Rapid Animal Genomic DNA Isolation Kit (Sangon Biotech, Shanghai, China). Libraries were prepared on an Illumina MiSeq PE300 platform (Sangon Biotech, Shanghai, China). Low-quality and short reads were removed using Fastp v. 0.36 (Chen et al. 2018) to obtain clean reads and ensure rich quality of information analysis.

### ﻿Mitogenome assembly, annotation, and analyses

SPAdes v. 3.15 (Bankevich et al. 2012) was used to assemble the high-quality next generation sequencing data de novo to construct contig and scaffold. After the assembly was completed, we evaluated and quality controlled the assembly results, excluding any contamination that may originate from the host genome in the subsequent analysis, and only retained the scaffolds derived from the genome of the organelle. We used BLASTn to compare the scaffolds with the NCBI library to obtain sequence similarity information, extracted the sequencing depth and coverage information of each scaffold, and manually selected possible target scaffolds after sorting out and comprehensively considering the above information. Then GapFiller v. 1.11 (Boetzer and Pirovano 2012) was adopted to supplement GAP to the contig obtained by splicing, and PrInSeS-G was adopted to carry out sequence correction to correct editing errors and insertion and deletion of small fragments in the splicing process, and finally the complete mitochondrial genome was obtained.

For mitochondrial gene annotation, we used tBLASTn and GeneWise to back-align with near-source reference databases to obtain the coding sequence (CDS) gene boundaries, and MiTFi to obtain the transfer RNA genes (tRNAs) sequence annotation. The non-coding ribosomal RNA genes (rRNAs) were identified by cmsearchrfam alignment and finally summarized into a complete annotation result.

The nucleotide composition and RSCU (relative synonymous codon usage) were calculated using PhyloSuite v. 1.2.2 (Zhang et al. 2020) and MEGA X (Kumar et al. 2018). Strand asymmetry was calculated using the formula: AT-skew = [A−T]/[A+T] and GC-skew = [G−C]/[G+C] (Perna and Kocher 1995). DnaSP v. 5 (Librado and Rozas 2009) was used to calculate the value of Ka (the nonsynonymous substitution rate), Ks (the synonymous substitution rate), and nucleotide diversity.

### ﻿Phylogenetic analyses methods

The mitochondrial genome data of 25 species in Pentatomomorpha were used to reconstruct the phylogenetic relationship of Lygaeoidea, in which 21 species of Lygaeoidea were regarded as ingroup and four species was regarded as outgroup (Table 1). All sequences were standardized and extracted 13 protein-coding genes (PCGs) by PhyloSuite v. 1.2.2 (Zhang et al. 2020). The 13 PCGs of the 25 species were aligned individually using codon-based multiple alignments with MAFFT v. 7.313 software with default settings (Katoh and Standley 2013). Gblocks v. 0.91b software was used to remove the intergenic gaps and ambiguous sites (Talavera and Castresana 2007), and all PCGs sequences were concatenated in PhyloSuite v. 1.2.2. The best partitioning scheme and evolutionary models for constructing Bayesian inference (BI) and maximum-likelihood (ML) trees were selected by PartitionFinder2 (Lanfear et al. 2016), with a greedy algorithm, BIC criterion, and the gene and codon model.

BI phylogenies were inferred using MrBayes v. 3.2.6 (Ronquist et al. 2012) under partition model (2,000,000 generations), in which the initial 25% of sampled data were discarded as burn-in. ML phylogenies were inferred using IQ-TREE (Nguyen et al. 2015) under the Edge-linked partition model for 5000 standard bootstraps with 1000 replicates.

## ﻿Results

### ﻿Genome structure and composition

**Figure 2.**
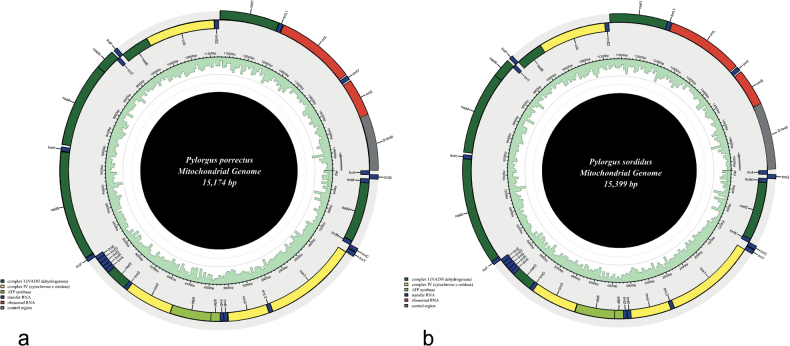
Circular maps of the complete mitogenome of *Pylorgus* species **a***P.porrectus***b***P.sordidus*. Different colors indicate different types of genes and regions. Genes in the outer circle are located on the J-strand, and genes in the inner circle are located on the N-strand.

The assembled complete mitogenomes of *Pylorgusporrectus* and *P.sordidus* are circular DNA molecules of 15,174 bp and 15,399 bp in length, respectively, which is within the range of the sequenced mitogenomes of Lygaeidae in GenBank (Table 1). These mitogenomes all have a similar typical insect mitogenome structure, closed-circular and double-stranded DNA, containing 13 PCGs, 22 tRNAs, two rRNAs, and a control region (D-loop) (Fig. 2). The sequence of mitochondrial protein-coding genes is the same as that in other Lygaeoidea (Cao et al. 2020). Among the 37 genes, 23 genes (9 PCGs and 14 tRNAs) are on the majority strand (N-strand), while the remaining four PCGs, eight tRNAs, and two rRNA genes are on the minority strand (J-strand).

**Table 2.** Base content of the mitochondrial genome.

Gene	Size (bp)	A	T	G	C	A+T%	AT-skew	GC-skew
*P.porrectus*	15,174	42.7	31.8	9.6	15.8	74.5	0.15	−0.24
*P.sordidus*	15,399	42.8	33.1	9.6	14.5	75.9	0.13	−0.2

The basic composition of *P.porrectus* was A = 42.7%, T = 31.8%, G = 9.6%, and C = 15.8%, and of *P.sordidus*, A = 42.8%, T = 33.1%, G = 9.6%, C = 14.5%. Furthermore, both mitochondrial genome sequences were biased toward A and T. The AT content of *P.porrectus* was 63.74% and that of *P.sordidus* was 64.12%. The AT-skew value was greater than 0, whereas the GC skew value was less than 0, indicating that the base composition of *P.porrectus* and *P.sordidus* showed a strong A-bias and T-bias (Table 2).

### ﻿Protein-coding genes

The complete length of the 13 PCGs of *P.porrectus* and *P.sordidus* were 10,991 bp and 10,993 bp, respectively. Of these, nine PCGs are located at the N-strand, and the other four PCGs were encoded on the J-strand (Fig. 2). Most PCGs started with ATN except for *cox1* and *nad4l* that began with TTG. Ten PCGs terminated with TAA/TAG, and the remaining three PCGs (*cox1*, *cox2*, and *cox3*) terminated with an incomplete T residue (Tables 3, 4). It has been speculated that these incomplete termination codons can be completed by adding ‘A’ during transcription (Ojala et al. 1981; Lavrov et al. 2002), and do not affect translation.

**Figure 3.**
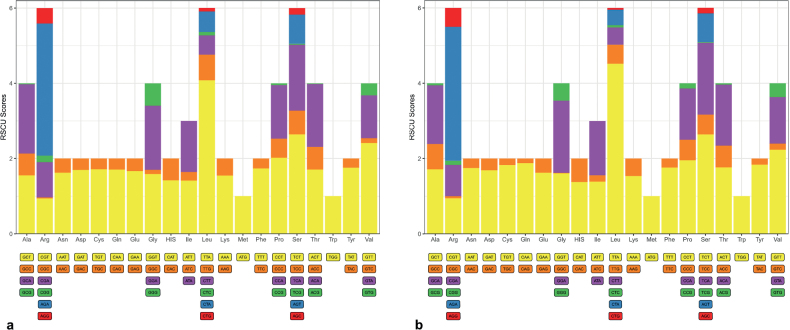
RSCU values of *Pylorgus* species **a***P.porrectus***b***P.sordidus*. The abscissa represents the type of amino acid translated by the codon, and the ordinate represents the codon bias score calculated for the amino acid. The higher the score, the more the types of codons, and the more active the evolutionary variation of genes in the genome.

The RSCU of the two species was calculated (Fig. 3). The codons that were most used TTA-Leu and AGA-Arg. Most of the frequently used codons are composed of A and T, which may be related to the fact that the A-T skewness is higher than the G-C skewness in the PCGs of the two species.

**Table 3.** Mitochondrial composition of *Pylorgusporrectus*.

Gene	Position (bp)	Size (bp)	Strand	Direction	Intergenic nucleotides	Anti- or start/stop codons
*trnI*	1–64	64	N	Forward	0	
*trnQ*	62–130	69	J	Reverse	−3	
*trnM*	131–200	70	N	Forward	0	
*nad2*	201–1187	987	N	Forward	0	ATT/TAA
*trnW*	1178–1241	64	N	Forward	−10	
*trnC*	1234–1296	63	J	Reverse	−8	
*trnY*	1303–1370	68	J	Reverse	6	
*cox1*	1374–2907	1534	N	Forward	3	TTG/T–
*trnL2*	2908–2972	65	N	Forward	0	
*cox2*	2973–3648	676	N	Forward	0	ATA/T–
*trnK*	3649–3713	65	N	Forward	0	
*trnD*	3714–3774	61	N	Forward	0	
*atp8*	3775–3933	159	N	Forward	0	ATA/TAA
*atp6*	3927–4592	666	N	Forward	−7	ATG/TAA
*cox3*	4601–5378	778	N	Forward	8	ATT/T–
*trnG*	5379–5443	65	N	Forward	0	
*nad3*	5444–5797	354	N	Forward	0	ATA/TAG
*trnA*	5796–5860	65	N	Forward	−2	
*trnR*	5861–5920	60	N	Forward	0	
*trnN*	5923–5990	68	N	Forward	2	
*trnS1*	5990–6058	69	N	Forward	−1	
*trnE*	6058–6121	64	N	Forward	−1	
*trnF*	6122–6184	63	J	Reverse	0	
*nad5*	6185–7882	1698	J	Reverse	0	ATT/TAA
*trnH*	7886–7949	64	J	Reverse	3	
*nad4*	7987–9303	1317	J	Reverse	37	ATG/TAA
*nad4l*	9297–9605	309	J	Reverse	−7	TTG/TAA
*trnT*	9581–9643	63	N	Forward	−25	
*trnP*	9644–9706	63	J	Reverse	0	
*nad6*	9709–10170	462	N	Forward	2	ATC/TAA
*cob*	10170–11306	1137	N	Forward	−1	ATG/TAG
*trnS2*	11305–11374	70	N	Forward	−2	
*nad1*	11396–12319	924	J	Reverse	21	ATC/TAA
*trnL1*	12320–12384	65	J	Reverse	0	
*rrnL*	12392–13612	1221	J	Reverse	7	
*trnV*	13635–13700	66	J	Reverse	22	
*rrnS*	13726–14315	590	J	Reverse	25	

**Figure 4.**
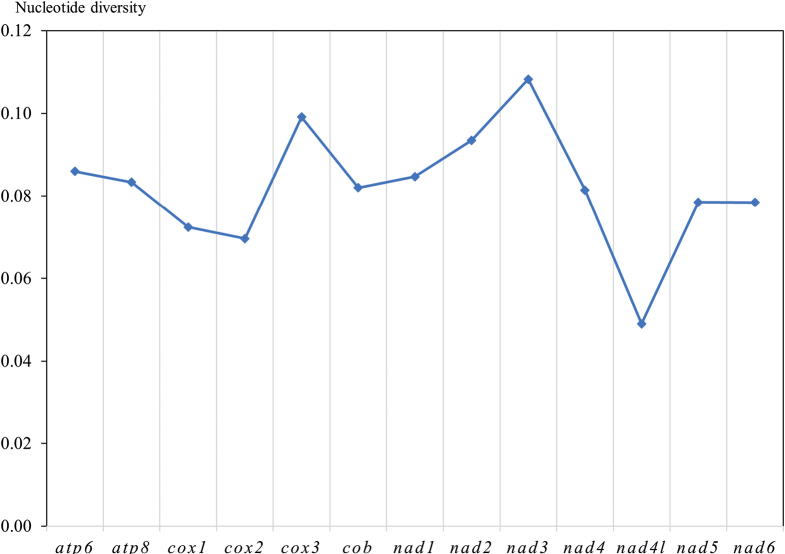
Nucleotide diversity (Pi) of 13 PCGs among two newly sequenced *Pylorgus* mitogenomes.

The nucleotide diversity (Pi) of the two species based on 13 PCGs was computed (Fig. 4) and ranged from 0.05 to 0.11. Among the PCGs, *nad3* (0.11) had the highest Pi values, and *nad4l* (0.05) had the lowest Pi values, which implies that *nad4l* is the most conserved gene in *Pylorgus*.

**Table 4.** Mitochondrial composition of *Pylorgussordidus.*

Gene	Position (bp)	Size (bp)	Strand	Direction	Intergenic nucleotides	Anti- or start/stop codons
*trnI*	1–64	64	N	Forward	0	
*trnQ*	62–130	69	J	Reverse	−3	
*trnM*	131–201	71	N	Forward	0	
*nad2*	202–1188	987	N	Forward	0	ATT/TAA
*trnW*	1179–1242	64	N	Forward	−10	
*trnC*	1235–1297	63	J	Reverse	−8	
*trnY*	1305–1373	69	J	Reverse	10	
*cox1*	1377–2910	1534	N	Forward	3	TTG/T–
*trnL2*	2911–2975	65	N	Forward	0	
*cox2*	2976–3651	676	N	Forward	0	ATA/T–
*trnK*	3652–3716	65	N	Forward	0	
*trnD*	3717–3777	61	N	Forward	0	
*atp8*	3778–3936	159	N	Forward	0	ATA/TAA
*atp6*	3930–4595	666	N	Forward	−7	ATG/TAA
*cox3*	4604–5381	778	N	Forward	8	ATT/T–
*trnG*	5382–5444	63	N	Forward	0	
*nad3*	5445–5798	354	N	Forward	0	ATA/TAG
*trnA*	5797–5860	64	N	Forward	−2	
*trnR*	5861–5920	60	N	Forward	0	
*trnN*	5923–5990	68	N	Forward	2	
*trnS1*	5990–6058	69	N	Forward	−1	
*trnE*	6058–6121	64	N	Forward	−1	
*trnF*	6122–6184	63	J	Reverse	0	
*nad5*	6185–7882	1698	J	Reverse	0	ATT/TAA
*trnH*	7886–7949	64	J	Reverse	3	
*nad4*	7988–9304	1317	J	Reverse	38	ATG/TAA
*nad4l*	9298–9606	309	J	Reverse	−7	TTG/TAA
*trnT*	9582–9644	63	N	Forward	−25	
*trnP*	9645–9707	63	J	Reverse	0	
*nad6*	9710–10171	462	N	Forward	2	ATC/TAA
*cob*	10171–11307	1137	N	Forward	−1	ATG/TAG
*trnS2*	11306–11375	70	N	Forward	−2	
*nad1*	11397–12320	924	J	Reverse	21	ATC/TAA
*trnL1*	12321–12385	65	J	Reverse	0	
*rrnL*	12397–13613	1217	J	Reverse	11	
*trnV*	13636–13701	66	J	Reverse	22	
*rrnS*	13727–14316	590	J	Reverse	25	

**Figure 5.**
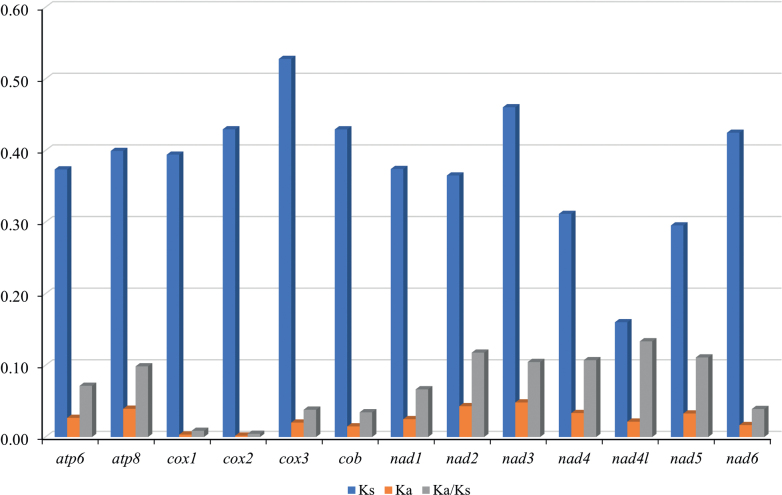
The ratios of Ka/Ks of 13 PCGs in the mitochondrial genomes of *Pylorgusporrectus* and *P.sordidus*.

The ratios of Ka/Ks for each gene of the 13 PCGs were also computed (Fig. 5). All Ka/Ks values were less than 1 and ranged from 0.01 to 0.13, indicating that the genes have been subjected to purification selection. In particular, the Ka/Ks values were the highest for *nad4* and *nad5*, suggesting that they had the highest evolution speed, and lowest for *cox1*, indicating the slowest evolution.

### ﻿Gene overlaps and intergenic spacers

Eleven gene overlaps were observed in the two mitogenomes, ranging from 1 bp to 25 bp (Tables 3, 4), and *nad4l* and *trnT* possessed the longest overlap.

Intergenic spacers were identified in the two mitogenomes, and their lengths ranged from 1 bp to 38 bp (Tables 3, 4). The longest intergenic spacer of 38 in *P.sordidus* was located between *trnH* and *nad4*.

### ﻿Transfer RNA and ribosomal RNA genes

The two mitogenomes both contain the complete set of 22 tRNA genes typical of Lygaeidae mitogenomes, ranging from 60 to 71 bp, which is consistent with previously sequenced mitogenomes of Lygaeidae (Cao et al. 2020; Huang et al. 2021). Fourteen of the 22 tRNAs were on the N-strand, and eight were on the J-strand (Fig. 2).

All tRNA have the typical cloverleaf secondary structure, including the TΨC arm, the amino acid acceptor arm, the anticodon arm, and the dihydrouridine arm. Some of tRNA genes (*trnY*, *trnA*, *trnS1*, *trnF*, *trnH*, *trnP*, and *trnV*) showed individual base mismatches, which is a common phenomenon in insect mitogenomes (Zhang et al. 2019).

The *rrnL* genes of the two mitogenomes are located at the intergenic region between *trnL* and *trnV*, with lengths that range from 1217 bp to 1221 bp. The *rrnS* genes are located between *trnV* and the D-loop, which are both 590 bp in length. Both rRNAs are located on the N-strand.

### ﻿Phylogenetic analysis

**Figure 6.**
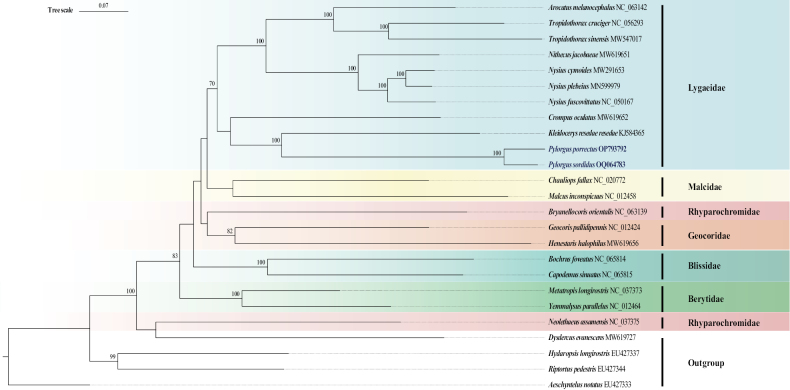
Phylogenetic tree inferred from ML methods based on 13 PCGs. Nodal support is given as standard bootstrap (%); only values > 70% are shown. The newly sequenced *Pylorgusporrectus* and *P.sordidus* mitogenomes are highlighted in dark blue and bold.

**Figure 7.**
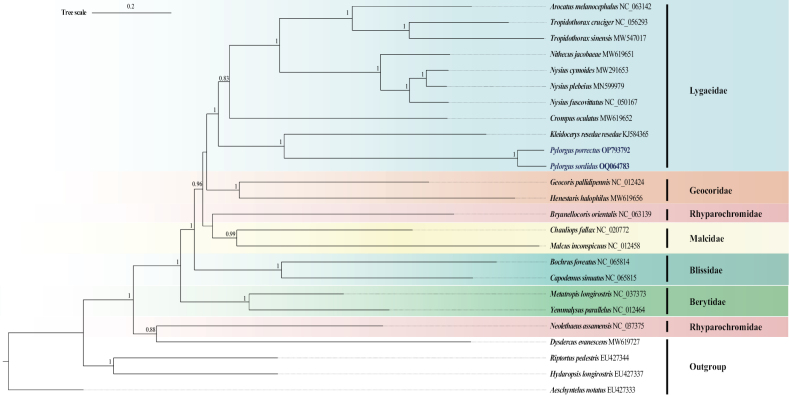
Phylogenetic tree inferred from BI methods based on 13 PCGs. Nodal support is given as partition model; only values > 0.80 are shown. The newly sequenced *Pylorgusporrectus* and *P.sordidus* mitogenomes are highlighted using dark blue and bold typeface.

Phylogenetic relationships within Lygaeoidea were reconstructed based on mitochondrial 13 PCGs using BI and ML methods (Figs 6, 7). A total of 21 Lygaeoidea species were selected as the ingroup and an additional four species from Pyrrhocoroidea, Coreoidea, Rhopalidae, and Alydidae were used as the outgroup. Compared to the ML tree, the BI tree had higher confidence values, and the monophyly of all the studied families was supported except Rhyparochromidae.

The clades making up the Lygaeidae had high support values in the BI results and confirmed the monophyly of Lygaeidae (Figs 6, 7). The monophyly of Lygaeidae was also supported in the ML results, but the nodal support is not so high. However, the Lygaeidae clusters as sister to Malcidae in the ML tree, but sister to Geocoridae in the BI tree, implying that the positions of Geocoridae and Malcidae are unstable. The two species of Rhyparochromidae are not clustered together. *Neolethaeusassamensis* clusters as sister to the Pyrrhocoroidea species, and together they are sister to the remaining ingroups.

## ﻿Discussion

In this study, we sequenced and analyzed the mitogenomes of *Pylorgusporrectus* and *P.sordidus*, which had common and similar structures. The mitochondrial genome structure of the two *Pylorgus* species is a double-stranded closed loop, containing a non-coding control region sequence and encoding 37 genes. The two species showed a substantial nucleotide bias toward a higher A and T content, as do other Pentatomomorpha (Zhang et al. 2019; Cao et al. 2020; Huang et al. 2021; Carapelli et al. 2021; Xu et al. 2021; Zhu et al. 2023). All PCGs began with ATN except for *cox1* and *nad4l* that started with TTG. In total, 10 PCGs terminated with TAA/TAG and the remaining three PCGs (*cox1*, *cox2*, and *cox3*) terminated with incomplete T residues. The calculation of Ka/Ks values revealed that *nad4* and *nad5* had relatively higher evolutionary rates, and *cox1* was determined to be the most conserved gene. Eleven gene overlaps were observed in the two sequenced mitogenomes, and gene overlaps have also been found in other known Lygaeidae mitogenomes (Cao et al. 2020). All tRNA molecules have a typical cloverleaf structure (Li et al. 2017).

The phylogenetic results using 13 PCGs confirm the monophyly of Lygaeidae, which support the opinions of Henry (1997), Henry et al. (2015), and Schuh and Weirauch (2020). The ML tree shows that the topology within Lygaeidae is Ischnorhynchinae + (Lygaeinae + Orsillinae) (Fig. 6; Table 1). This result is in agreement with Cao et al. (2020) and Carapelli et al. (2021) but differs slightly from Henry’s (1997) morphological hypothesis of Lygaeinae + (Ischnorhynchinae + Orsillinae). However, in the ML tree, *P.porrectus* and *P.sordidus* cluster with *Kleidocerysresedae* and then *Crompusoculatus* of Ischnorhynchinae (Fig. 6; Table 1), whereas in the BI tree, *P.porrectus* and *P.sordidus* only cluster with the *K.resedae*, and *C.oculatus* clusters with the other species of Lygaeinae and Orsillinae (Fig. 7; Table 1). We think this is mainly because the limited number of published mitogenomes within the Lygaeidae. This problem could be solved by sequencing additional mitogenomes of lygaeid species. The two selected species of Rhyparochromidae are not clustered together, which is similar with the results of Cao et al. (2020), Carapelli et al. (2021), and Huang et al. (2021). *Neolethaeusassamensis* clusters sister to the Pyrrhocoroidea species, and they together sister to the remaining ingroups in our result. More mitochondrial genomes need to be determined to better understand the monophyly of Rhyparochromidae. Overall, our results enrich the understanding of mitochondrial genome structure in the Lygaeidae and further supports the monophyly of the family containing the three subfamilies Ischnorhynchinae, Lygaeinae, and Orsillinae.

## ﻿Additional information

### Author ORCIDs

Cuiqing Gao https://orcid.org/0000-0002-0177-5161

Wen Dong https://orcid.org/0009-0004-6559-808X

### Data availability

All of the data that support the findings of this study are available in the main text or Supplementary Information.
